# Immunohistochemical Evaluation of p53 and Its Association With Prognostic Parameters in Breast Cancer

**DOI:** 10.7759/cureus.102798

**Published:** 2026-02-01

**Authors:** KN Kusuma, Shetty Shilpa Madhav, Shankar S Vijay

**Affiliations:** 1 Department of Pathology, Adichunchanagiri Institute of Medical Sciences, Adichunchanagiri University, Mandya, IND; 2 Department of Pathology, Subbaiah Institute of Medical Sciences, Shimoga, IND

**Keywords:** breast carcinoma, immunohistochemistry, ki-67, molecular subtype, p53

## Abstract

Background

P53 is a tumour suppressor gene that is commonly altered in breast cancer, potentially affecting tumour behaviour and prognosis. However, the clinicopathological and molecular associations vary across studies. Hence, the present study aims to evaluate p53 positivity and its association with clinicopathological parameters and molecular subtypes in invasive breast carcinoma.

Aims and objectives

To evaluate p53 positivity in invasive breast carcinoma and analyse its association with key clinicopathological parameters.

Methods

A cross-sectional observational study was undertaken in the pathology department of a tertiary care hospital. Immunohistochemistry was used to measure p53 expression, which was then linked with clinicopathological features like tumour size, histological grade, lymph node status, lymphovascular invasion, hormone receptor status, human epidermal growth factor receptor 2 (HER2) status, Ki-67 proliferation index, and molecular subtype. Statistical analysis was done using SPSS software. Chi-square and Fisher's exact tests were used for correlation analysis, and p-values <0.05 were considered significant.

Results

P53 positivity was detected in 57.5% of the cases. P53 positivity was shown to be significantly associated with greater tumour size (p=0.029) and a high Ki-67 index (p=0.042) whereas histological grade, lymph nodal metastasis, lymphovascular invasion or hormone receptor status did not show statistical significance. Although hormone receptor-negative tumours showed more frequent p53 positivity status compared to a negative status, the results were not significant (p=0.052). P53 positivity was most frequent in aggressive molecular subtypes of tumours, like HER2-enriched (77.8%) and luminal B (75%) tumours.

Conclusion

P53 expression is associated with aggressive clinicopathological features like large tumour size, high Ki-67 index and the HER2-enriched subtype, suggesting its role in tumour progression and aggressiveness. Further, large multicentric studies with survival analysis are needed to confirm its clinical validity.

## Introduction

Breast cancer is the most commonly diagnosed malignancy and is one of the leading causes of cancer-related death in women globally. It is a biologically heterogeneous disease with a wide range of clinical characteristics and therapeutic responses [1]. Treatment decisions are still influenced by conventional prognostic indicators such as tumour size, histological grade, lymph node status, and hormone receptor positivity (estrogen receptor (ER)/progesterone receptor (PR)) [2]. However, in order to capture the biological diversity of breast cancer and enable more accurate risk categorization, the incorporation of molecular markers has become crucial.

P53, one of the well-known tumor suppressor genes, has been subjected to research. This gene is located on chromosome 17p13.1 and maintains the genomic integrity of the cell by controlling the cell cycle, repairing the DNA, and inducing the apoptosis in response to genotoxic stress [3].

Nearly 20%-30% of breast cancer shows p53 mutation, which facilitates tumour growth by a dual mechanism: loss of tumour suppression activity and gaining the oncogenic activity. The most common type of mutation is missense mutation, which accounts for 90% of the cases. This missense mutation leads to dysfunctional mutant p53 protein that has increased stability, which leads to its accumulation in the nucleus, and this can be detected by immunohistochemistry (IHC). Hence, IHC can be used as an alternative to detect the presence of these mutations. Null mutation is less common and leads to nonfunctional mutated protein and is usually not detected by IHC [4,5].

The prognostic value of p53 positivity by IHC remains controversial, especially in triple-negative breast cancers. Some studies reported the aggressive and metastatic phenotype with the worst outcome [6,7]. While other studies suggested that p53 positivity is due to wild-type p53 protein, which is overexpressed as a result of a compensatory mechanism to repair the DNA that is damaged during the tumour development, hence relating it to a favourable outcome [8,9]. Also, there is no validated cutoff for p53 expression; 10% and 50% are the most commonly used cutoffs [10].

Hence, the p53 expression by IHC is not yet clinically validated, and its expression is still debatable. Therefore, the present study aims to evaluate the p53 expression in breast carcinoma and to correlate the expression with various clinicopathological factors and molecular subtypes.

## Materials and methods

This prospective observational study was carried out in the Department of Pathology at a tertiary care hospital after obtaining ethical approval from the Institutional Ethics Committee (AIMS/IEC/05/2022) and reported according to STROBE (STrengthening the Reporting of OBservational studies in Epidemiology) guidelines. We included a total of 40 cases of invasive breast cancer over a period of three years. Only cases with modified radical mastectomy with complete clinical information were selected. Patients who had undergone chemotherapy or radiotherapy prior to surgery, as well as cases involving lumpectomy and core biopsy, were excluded from the study.

After receiving the surgical specimens, relevant clinical details were collected from patient records, and tissues were fixed in formalin, processed, and embedded in paraffin blocks. Haematoxylin and eosin (H&E) stained slides were prepared for microscopic examination. Tumours were graded according to Nottingham’s grading, and the presence of lymphovascular invasion and metastasis was recorded [2].

All the cases were subjected to IHC for ER, PR, HER2, Ki-67, and p53, along with suitable controls. ER and PR were considered positive when ≥1% of nuclei were stained [11]. HER2 was evaluated according to American Society of Clinical Oncology/College of American Pathologists (ASCO/CAP) criteria, with a 3+ score considered positive and 0 and 1+ as negative, and equivocal cases were confirmed by fluorescence in situ hybridization (FISH) [12]. A Ki-67 index of ≥20% was considered high [13].

For p53 IHC, a monoclonal antibody from Biocare Medicals (Pacheco, CA, USA) with a dilution of 1:100 was used. Heat-induced antigen retrieval was done using ethylenediamine tetraacetic acid (EDTA) buffer, and immunodetection was done using a polymer-based horseradish peroxidase (HRP) detection system with 3,3'-diaminobenzidine (DAB) as a chromogen. For scoring, a combined score of the percentage of nuclei stained along with intensity was used. A known p53-positive carcinoma tissue was used as a positive control. Negative control was obtained by omitting the primary antibody. Additionally, stromal cells were used as an internal negative control. Nuclear positivity was considered for scoring. P53 was considered positive if more than 10% of cells showed intermediate to strong staining and considered negative if less than 10% of cells showed weak staining or no staining at all [14]. The 10% cut-off was chosen because it is the most often used threshold in the literature on breast cancer and reduces false positivity from basal p53 expression while maintaining sensitivity for mutant instances.

Further, the tumours were classified into molecular subtypes: luminal A (ER/PR positive, HER2 negative, Ki-67 <20%), luminal B (ER/PR positive, HER2 positive or negative, Ki-67 ≥20%), HER2-enriched (ER/PR negative, HER2 positive), and triple-negative (ER, PR, and HER2 negative) [15].

Finally, the p53 status was correlated with clinicopathological factors and molecular subtypes.

Statistics

Data were analysed using IBM SPSS Statistics, version 25.0 (IBM Corp, Armonk, NY). The data were summarised using descriptive statistics, which included means and standard deviations for continuous variables and frequencies and percentages for categorical variables. The chi-square test or Fisher's exact test, depending on the data distribution, was utilised for evaluating associations between p53 and clinicopathological features such as tumour size, grade, lymphovascular invasion, and nodal status along with IHC markers like ER, PR, HER2 neu, and Ki 67. A p-value of <0.05 is considered statistically significant.

## Results

Patients’ characteristics

We evaluated a total of 40 cases of invasive breast carcinoma, which we received during the study period. Grade 2 tumours were predominant, accounting for 60% of cases. With respect to size, the T2 stage was most predominant (26 cases). Lymph node involvement and lymphovascular invasion were seen in 22 cases, constituting 55% each (Table 1). Luminal A was the most commonly encountered molecular subtype, constituting 35% of the cases, followed by Luminal B (30%), HER2-enriched (22.5%) and triple-negative tumours (12.5%).

**Table 1:** Association of p53 with Clinicopathological and Immunohistochemical Features N is the total number of cases used for calculating the percentages for individual variables. ER: Estrogen receptor; PR: progesterone receptor; HER2: human epidermal growth factor receptor 2.

Variables	p53 Negative	p53 Positive	Total cases (N=40)	Statistical test used	P value
Tumour grade					
Grade 1 (N=7)	4 (57.1%)	3 (42.9%)	7 (17.5%)	Fisher’s exact test	0.327
Grade 2 (N=24)	11 (45.8%)	13 (54.2%)	24 (60%)		
Grade 3 (N=9)	2 (22.2%)	7 (77.8%)	9 (22.5%)		
Tumour size					
T1 (N=6)	1 (16.6%)	5 (83.3%)	6 (15%)	Fisher’s exact test	0.029
T2 (N=26)	15 (57.7%)	11 (42.3%)	26 (65%)		
T3 (N=8)	1 (12.5%)	7 (87.5%)	8 (20%)		
Lymph node involvement					
Negative (N=22)	8 (36.4%)	14 (63.6%)	22 (55%)	χ² = 0.753	0.381
Positive (N=18)	9 (50%)	9 (50%)	18 (45%)		
Lymphovascular invasion					
Negative (N=22)	10 (44.5%)	12 (54.5%)	22 (55%)	χ²=0.174	0.676
Positive (N=18)	7 (38.8%)	11 (61.2%)	18 (45%)		
ER/PR					
Negative (N=14)	5 (35.7%)	9 (64.3%)	14 (35%)	χ²=0.408	0.524
Positive (N=26)	12 (46.2%)	14 (53.8%)	26 (65%)		
HER2 neu					
Negative (N=23)	12 (52.2%)	11 (47.8%)	23 (57.5%)	χ²=2.07	0.149
Positive (N=17)	5 (29.4%)	12 (70.6%)	17 (42.5%)		
Ki 67 index					
Low (N=13)	9 (69.2%)	4 (30.8%)	13 (32.5%)	Fisher’s exact test	0.042
High (N=27)	8 (29.7%)	19 (70.3%)	27 (67.5%)		

Correlation of p53 with clinicopathological features

**Figure 1:**
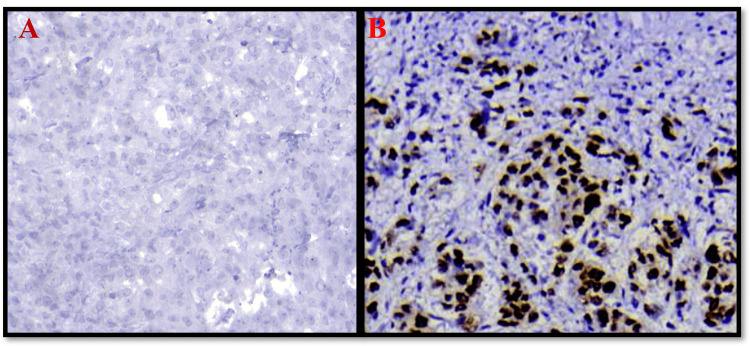
Photomicrograph of immunohistochemical stain of p53 40x (A: negative; B: positive)

Out of 40 cases studied, p53 was positive in 23 cases, accounting for 57.5%, and negative in 17 cases (42.5%) (Figure 1). On correlating the size of the tumour with p53, out of the total cases of T3-stage tumours, seven cases showed p53 positivity (87.35%), whereas only 42.3% of cases of tumours in T2 showed p53 passivity. These results were statistically significant with a P value of 0.029.

With respect to tumour grade, the results showed the increasing trend of p53 with higher grade: 42.5% in grade 1, 54.1% in grade 2 and 77.7% in grade 3. However, results were not statistically significant (p=0.327). Similarly, p53 positivity was higher in lymphovascular invasion cases (61%) but with no statistically significant results, whereas lymph node-positive cases showed lower p53 positivity, which also did not show any significant association (Table 1).

Correlation of p53 with molecular profile

Among the ER/PR-negative cases, nine (64.3%) showed p53 positivity, compared to 14 (53.8%) of ER/PR-positive cases. However, the association between ER/PR status and p53 positivity was not statistically significant (p>0.05).

Regarding HER2 status, 17 (42.5%) tumours were HER2 positive. Among them, 12 (70.6%) were p53 positive, while 11 (47.8%) of the HER2-negative tumours were p53 positive. Although p53 positivity appeared more frequent in HER2-positive tumours, the association was not statistically significant (p=0.264).

In contrast, Ki-67 showed a significant association with p53 expression. Of the 27 tumours with a high Ki-67 index, 19 (70.4%) were p53 positive, whereas only four (30.8%) of the 13 low Ki-67 tumours were p53 positive. These findings were statistically significant with a p-value of 0.042, suggesting a positive correlation between p53 positivity and proliferative index.

**Figure 2:**
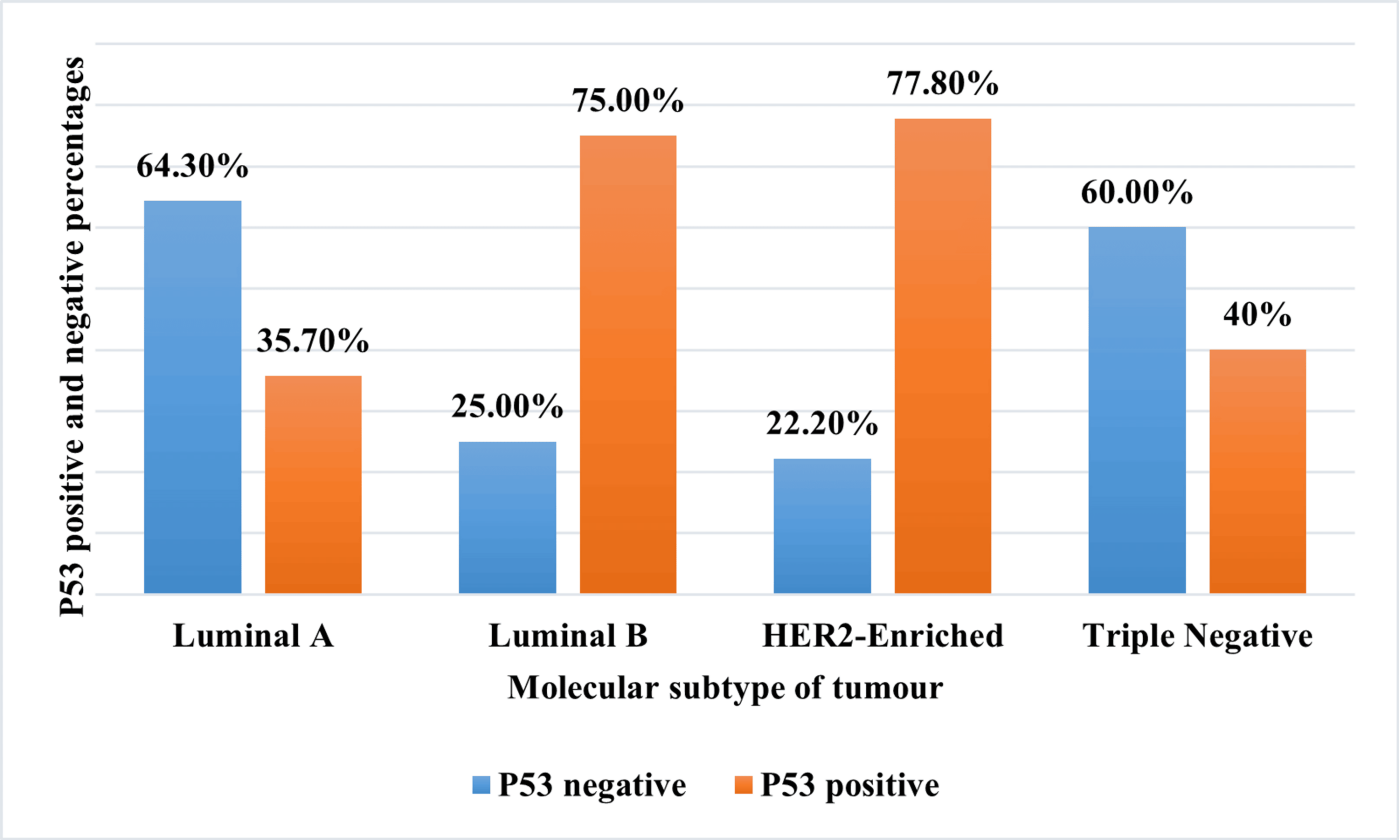
Graph showing the p53 status in molecular subtypes of tumours. HER2: human epidermal growth factor receptor 2.

P53 positivity was more in HER2-enriched (77.8%) and luminal B (75%) tumours compared to luminal A (35.7%) and triple-negative tumours (40%). However, the results did not reach statistical significance (Figure 2). This variation might be due to a smaller sample size and non-uniform distribution of tumours in different subtypes.

## Discussion

The p53 gene, first identified in 1979, was initially thought to be an oncogene; however, it was later recognised as a tumour suppressor gene [3]. The p53 gene is one of the most frequently mutated genes identified in human neoplasia, with mutations predicted to occur in up to 50% of all malignancies [16].

In our study, most of the cases (57.5%) showed p53 positivity; this was similar to studies by Patnayak et al., Song et al., Sekar et al. and Dash et al. [17-20]. In contrast, studies by Radha et al., Al-Joudi, Rashed et al., Abdollahi et al., and Neharika et al. showed lower p53 positivity of 22%, 29.6%, 34%, 37.4%, and 47.4%, respectively [21-25]. These discrepancies in p53 expression in various studies are due to the use of different antibodies, different cutoffs for interpretation and different staining protocols used for IHC. For instance, some studies used a cutoff of 10% for interpretation, while others used 50%. Hence, it is difficult to compare the studies directly and to draw a conclusion [10,26]. Therefore, standardised criteria should be made for interpretation in order to utilise p53 IHC as a surrogate marker for gene expression.

We found a strong association of p53 with tumour size stage, with larger tumours showing a higher percentage of p53 positivity, which was consistent with the study by Neharika et al. [25]. In contrast, studies by Song et al., Sekar et al., and Dash et al. showed no significant association [18-20].

The study did not show statistically significant results with respect to tumour grade; however, there was an increasing trend of p53 positivity towards higher-grade tumours, i.e., 42.9%, 54.2% and 77.8% in grade 1, grade 2 and grade 3 tumours, respectively. Similar findings were observed in studies by Song et al., Dash et al. and Arora et al. [18,20,26]. In contrast, studies by Al-Joudi, Gupta et al. and Yamashita et al. showed a statistical association [22,27,28]. The observed trend in our study supports the hypothesis of more frequent p53 alteration in poorly differentiated tumours.

Similarly, there was no association between lymphovascular invasion and p53, which aligns with studies by Al-Joudi, Arora et al. and Piplani et al. [22,26,29]. This was in contrast to studies by Neharika et al. and Gupta et al. [25,27]. Interestingly, we had more p53-positive cases in lymph node-negative cases compared to lymph node-positive cases (63.6% vs 50%). However, there was no significant association found, similar to studies by Arora et al., Piplani et al. and Al-Joudi [22,26,29].

Although the p53 expression did not show a statistically significant association with hormone receptors, the trend showed that expression increased towards the hormone-negative cases (64.3% vs 53.8%), similar to studies by Arora et al. and Neharika et al. [25,26]. This reflected the inverse relationship between p53 expression and hormone receptor, suggesting the contribution of p53 abnormality to the aggressive behaviour of the tumour. In contrast, studies by Al-Joudi et al. and Piplani et al. did not show any correlation.

About 70.6% of HER2-positive cases showed p53 positivity; in contrast, only 47.8% of HER2-negative tumours showed p53 positivity. However, there was no statistically significant association similar to studies by Arora et al. and Abdollahi et al. [24,26]. In contrast, studies by Neharika et al., Patnayak et al. and Rashed et al. showed a positive association, suggesting the coexistence of HER2 and p53 expression as a poor prognostic marker [17,23,25].

We found a significant association between p53 and Ki 67 expression, with high Ki-67 index tumours showing more p53 positivity compared to low Ki index cases (70.4% vs 30.8%). This association suggested the role of p53 in controlling the uncontrolled proliferation of cells, thereby contributing to tumour growth and aggressiveness. These findings were similar to studies by Dash et al. and Madani et al. [20,30].

Further molecular subtyping of tumours revealed that more aggressive tumours, like HER2-enriched and luminal B tumours, had more p53 positivity of 77.8% and 75%, respectively. We had only five cases of triple-negative tumour, out of which only two had p53 positivity, constituting 40%, which was low compared to studies by Dash et al., who reported the higher percentage [20]. Luminal A tumours, which have a better prognosis, had a lower percentage of p53 positivity of 35.7%. The observed finding in our study reinforces the role of p53 in tumour aggressiveness.

Our study showed the positive association of p53 with aggressive tumour features like tumour size and Ki67 index. Although there was no statistical association between the other aggressive parameters, like tumour grade, lymphovascular invasion and molecular subtypes, the trend of increasing p53 positivity towards the aggressive features suggested the role of p53 in predicting tumour aggressiveness. The lack of association could be due to the smaller sample size and heterogenicity of breast tumours. In addition, the inclusion of region-specific data will add to existing gaps in the literature.

Our major limitations of the study are the small sample size and the single-centre study, and we did not do survival analysis. In addition, we could not compare the IHC with mutational analysis that would add more advantage. Hence, large multicentric study with survival analysis and comparison with the mutational analysis is needed to clarify the prognostic value of p53.

## Conclusions

P53 plays an important role in determining the biological behaviour of invasive breast carcinoma, and also it is related to tumour aggressiveness and progression. Variability in p53 expression reported by various studies underscores the need for standardised immunohistochemical interpretation criteria. Despite limitations such as a relatively smaller sample size, lack of survival and mutation analysis data, the present study provides region-specific data and lays a foundation for further large multicentric studies with survival analysis and molecular profiling.
